# Missed opportunities in family planning: process evaluation of family planning program in Omo Nada district, Oromia region, Ethiopia

**DOI:** 10.1186/s40834-018-0068-7

**Published:** 2018-08-01

**Authors:** Misganu Endriyas, Tefera Belachew, Berhane Megerssa

**Affiliations:** 1 Health Research and Technology Transfer Support Process, SNNPR Health Bureau, Hawassa, Ethiopia; 2 Department of Population and Family Health, Jimma University, Jimma, Ethiopia; 3 Department of Health Economics, Management and Policy, Jimma University, Jimma, Ethiopia

**Keywords:** Process evaluation, Family planning, Service quality, Missed opportunities, Omo Nada, Oromia, Ethiopia

## Abstract

**Background:**

Family planning (FP) program is a key program to avert unbalanced human population growth, maternal mortality, unintended pregnancy, unsafe abortion, sexually transmitted diseases and malnutrition. To address these aims, all services that clients receive must be of consistently high quality. So, services that clients receive should be monitored and evaluated.

**Methods:**

Case study was carried out in January, 2011, in Omo Nada district, Oromia region. Data were collected using different data collection methods. Process of FP program was evaluated using Judith Bruce model. Geographical accessibility, availability of resources for service provision and technical compliance were assessed. Level of program implementation was measured using stakeholders’ agreed indicators and judgment matrix.

**Results:**

Though overall program implementation level was good and clients were satisfied, notable gaps observed were absence of crucial materials, poor provision of information in relation to method given, poor technical performance in following aseptic procedure, and poor integration of services.

**Conclusion:**

Service provision should be monitored to maintain quality of service by integrating available services in resource limited setting.

**Electronic supplementary material:**

The online version of this article (10.1186/s40834-018-0068-7) contains supplementary material, which is available to authorized users.

## Background

Family planning (FP) is a key intervention for improving the health of women, men and children [1, 2]. Contraceptives prevent unintended pregnancies, reduce number of abortions, and lower incidence of death and disability related to complications of pregnancy and childbirth [3]. As an important component of reproductive health (RH), quality FP is recognized as a human right. All individuals have right to access, choice, and benefits of scientific progress in the selection of FP methods [4].

Despite decades of international agreements declaring the need for urgent action to improve wellbeing among women and newborns, deaths and poor health among these groups have remained high in developing world [5]. In developing countries, more than 120 million couples have an unmet need for safe and effective contraception. Out of 182 million pregnancies occurring each year in the developing world, about 40% are unwanted or ill-timed [6]. In 2008, an estimated 41% of all pregnancies in Ethiopia were unintended; 48% of all pregnancies in Oromia region were unintended and 76% of reproductive age of this region had unmet need to modern contraceptives [7].

Ethiopian ministry of health, with respect to improving maternal and child health, had planned to increase contraceptive prevalence rate to 66% by 2015. In order to achieve this target, the ministry had given priority to the provision of safe motherhood services such as family planning in the community [8]. But according to Ethiopian Demographic and Health Survey (2011), modern contraceptive prevalence rate was 27.3 nationally and 24.9 in the study region which was below national average [9].

The outcome of women health in developing countries is very low due to different socio-economic, socio-cultural factors, and lack of healthcare services. More specifically, restrictions on information about sexuality and contraception limit people’s ability to make choices regarding their own sexual and RH and rights [10].

For FP to meet its goals, all services that clients receive must be of consistently high quality and services that clients receive should be monitored and evaluated. Successful delivery of FP services requires proper coordination of activities that are involved at the various steps of the service delivery chain: counseling, provision of a wide choice of contraceptives, follow-up and appropriate referral, supervision, monitoring and evaluation, and a functional logistics system [11]. The purpose of this evaluation was to assess the program operations to highlight gaps to improve program implementation.

## Methods

Facility based descriptive case study was conducted in Omo Nada district in January, 2011. Case study was used because it lets to focus on selected area, in-depth understanding of issue and collecting data in different ways [12, 13]. Omo Nada district is one of rural districts in Oromia region, Ethiopia. In 2011, the district had four health centers. We considered two health centers (HCs) (Asendabo and Nada) purposely as the rest two were relatively new.

Building on work of Avedis Donabedian [14], Judith Bruce developed a frameworks for assessing quality of FP services [15]. We used Bruce’s framework as it provides a comprehensive framework for evaluating interpersonal dimension of quality of care and for developing appropriate indicators. The Bruce framework consists of six main elements: choice of methods, information to clients, technical competence, interpersonal relations (the degree of empathy; trust and confidentiality), mechanisms for encouraging continuity and appropriate constellation of services. In addition to availability of contraceptives, availability of other materials and trained provider were assessed as these resources are crucial to provide quality service [16].

Different data sources and data collection methods (client exit interview, observation and provider interview) were used. Data collection tools were adapted from standard tools [17, 18], translated to local language (Afan oromo for exit interview and Amharic for provider interview) and pre-tested.

We interviewed clients of FP service over one month study period. Exit interviews were completed by unemployed nurses who had diploma and speak local language. Observation of counselling session of new clients or clients continuing after discontinuation were done by nurses and health officers who had bachelor degree and working in other health center. Semi-structured questionnaire was self-administered to interview providers who had more than six months experience. To get more about data sources and items measured, see additional file (Additional file 1).

Evaluation is most effective, meaningful and useful when it is conducted with stakeholders using participatory and learning-oriented approaches. Involving stakeholders in evaluation processes contributes to building their own capacity to do future evaluation work [19, 20]. For this reason, program evaluation indicators and measurement matrix were prepared as operational definition in agreement with program stakeholders and are available in additional file (Additional file 1). Even though stakeholders of family planning program are diverse, we included program implementers (program officers and service providers) to develop performance measurement matrix. The overall findings were summarized and compared with preset performance judgment criteria to judge the level of achievement (Table 1 and Additional file 1).

**Table 1:** Overall judgment matrix for evaluating the level of implementation, Omo Nada district, 2011

Performance criteria	Performance level
≤ 25%	No implementation
26–50%	Critical
51–65%	Acceptable
66–80%	Good
81–90%	Very good
91–100%	Excellent

Data were entered in to Epi-data entry version 3.1 and exported to SPSS for windows version 16 for data analysis. Descriptive statistical tests were used to describe study variables. Qualitative data were analyzed manually by coding, categorizing, and thematising.

Ethical clearance was obtained from Jimma University College of Public Health and Medical Science Ethical Committee. Verbal consent was obtained from all respondents (both provider and client for observation) after through explanation of study objective. Data were analyzed anonymously and confidentiality was guaranteed.

## Results

A total of 181 clients were interviewed (103 from Nada and 78 from Asendabo HC) over one month. Half of clients (50.3%) were unable to read and write and about two fifths (38.7%) were in age range of 15–20 (with mean of 23.99 and SD = 5.26). Majority of clients (91.2%) were married and 76.2% had 1–4 living children (Table 2).

**Table 2:** Socio-demographic characteristics of clients using FP program in Omo Nada district, 2011

Socio-demographic characteristics		Frequency (*n* = 181)	Percent
Age	15–20	70	38.7
	21–25	50	27.6
	26–30	45	24.9
	31–35	13	7.2
	> 35	3	1.6
Educational background	Can’t read and write	91	50.3
	1–4	18	9.9
	5–8	39	21.5
	9–10	20	11.1
	> 10	13	7.2
Ethnicity	Oromo	146	80.7
	Amhara	19	10.5
	Yem	13	7.2
	Hadiya	2	1.1
	Other	1	0.5
Religion	Muslim	125	69.1
	Orthodox	49	27.1
	Protestant	7	3.8
Occupation	Farmer	99	54.7
	Merchant	49	27.1
	Student	14	7.7
	Employed	17	9.4
	Other	2	1.1
Marital status	Married	165	91.2
	Single	12	6.6
	Divorced	3	1.7
	Widowed	1	0.5
Monthly income	< 250	89	49.2
	251–500	58	32.0
	501–750	19	10.5
	751–1000	14	7.7
	> 1000	1	0.6
Desire to have number of children	Not decided	48	26.5
	1–4	53	29.3
	> 4	80	44.2
Living children	No children	24	13.3
	1–4	138	76.2
	> 4	19	10.5
Total		181	100

A total of 30 client-provider interaction sessions (15 from each HC) were observed from which 25 sessions were counseling of new clients while 5 were counseling of clients continuing after discontinuation. Majority, 28 (93.3%), of sessions were provided by female providers. Most sessions, 22 (73.3%), were provided by clinical nurses and rest 8 (26.7%) by mid-wife nurses.

Majority of providers (8 out of 10) interviewed were clinical nurses and all were diploma holders. Mean age of providers was 27.5 years (SD = 7.35) (Table 3).

**Table 3:** Socio-demographic characteristics of providers working in HCs in Omo Nada district, 2011

Characteristics		Frequency	Percent
Sex	Male	6	60
	Female	4	40
Age	20–30	8	80
	31–40	1	10
	> 40	1	10
Educational background	Diploma	10	100
Ethnicity	Oromo	7	70
	Amhara	1	10
	Other	2	20
Religion	Muslim	4	40
	Orthodox	6	60
Marital status	Married	7	70
	Single	3	30
Profession	Clinical Nurse	8	80
	Mid-wife	2	20
Work experience (year)	0.5–2	5	50
	2.1–10	3	30
	> 10	2	20
	Total	10	100

### Availability

We assessed availability of 6 choices of contraceptives (pills, Depo-Provera, implant, condom, emergency contraceptive and natural method counselling), trained provider, 13 amenities and materials (running water, waiting room, toilet, private room, hand washing water, soap, gloves, sharp box, height-weight scale, examination table, disinfectant, speculum and BP apparatus) and 5 IEC materials and guidelines (posters, sample contraceptives, flip charts, clinical guidelines and any other guidelines). Four contraceptive options, trained provider, 9 amenities and materials and 2 IEC materials were seen. This yielded 64% for measuring availability of materials for service provision (Additional file 1).

### Information provided

**Fig. 1:**
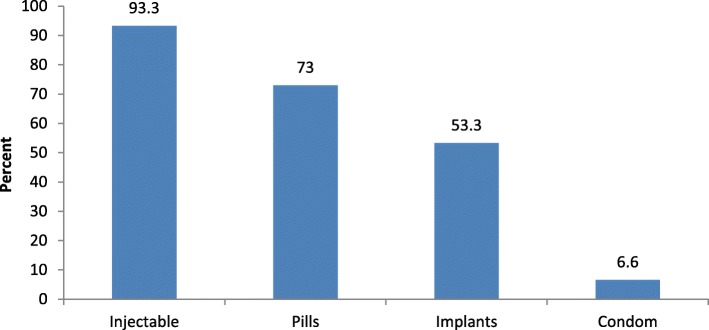
Methods discussed with clients in HCs, Omo Nada district, 2011

During observation, majority of clients, 28 (93.3%), were informed about available methods, decided and received method of their choice. The same proportion (93.3%) of clients were informed about injectable while emergency contraceptive and natural method counseling were not discussed in both HCs (Fig. 1).

### Choice of method

**Fig. 2:**
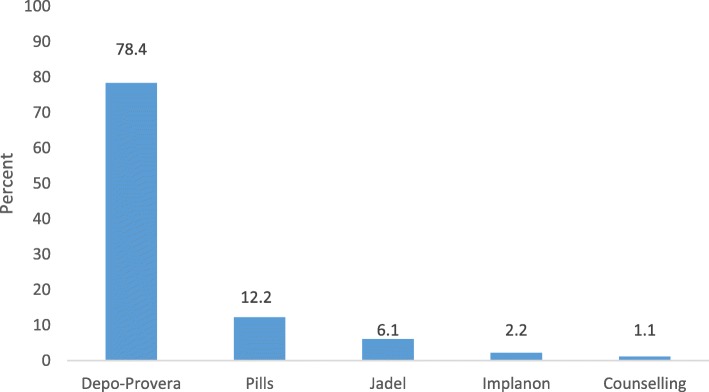
Contraceptives methods used by clients in HCs, Omo Nada district, 2011

From 181 respondents, 179 (98.9%) received contraceptives while 2 clients received only counselling on reproductive health. Majority of clients, 177(97.8%), used contraceptives of their choice, most preferred Depo-Provera (Fig. 2).

**Fig. 3:**
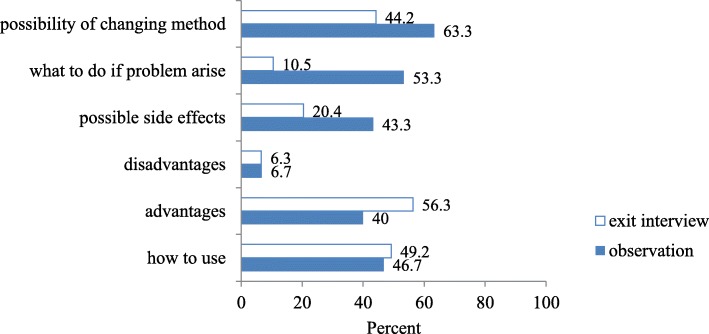
Comparison of information provided with observation and exit interview, Omo Nada district, 2011

Basic information that should be provided for clients in relation to chosen method were assessed using client exit interview and observation. Except advantages and how to use, all other information provided was higher with observation but both data collection methods showed that information regarding HIV prevention was missed (0%) (Fig. 3).

### Interpersonal relation

To measure interpersonal relation, clients feeling about expressing ideas freely and privacy during counselling and examination were assessed. About two thirds, 116 (64.1%), of clients said that they expressed their ideas freely while 30 (16.6%) complained that privacy was not maintained.

### Technical competence

During observation, thirty five activities and counselling (from welcoming to giving appointment) were assessed. The assessment showed that some of key activities/counselling like checking weight, height and assessing history of STI/s were not done for all observed cases while blood pressure was measured only for 2 (6.7%) cases. All clients were encouraged to continue using FP by discussing return visit and giving appointment. Overall measurement of technical competence gave 167.1/350 (Table 4).

**Table 4:** Technical competence during FP counselling in HCs, Omo Nada district, 2011

Activities	Percent (*n* = 30)	Result out of 10
1. Greet client in a friendly manner	24(80%)	8.0
2. Encourage client to ask questions	22(73.3%)	7.3
3. Treat client with respect	28(93.3%)	9.3
4. See client in private	22(73.3%)	7.3
5. Use visual aids	9(30%)	3.0
6. Use client’s records	30(100%)	10
7. Explicitly mention that the condom protects against STIs/HIV/AIDS	4(13.3%)	1.3
8. Previous contact with provider	24(80%)	8.0
9. Current age	24(80%)	8.0
10. Marital status	26(86.7%)	8.7
11. Whether sexually active or abstinent	9(30%)	3.0
12. Whether partner had more than one sexual partner in last year	0(0%)	0.0
13. Date of last delivery	8(26.7%)	2.7
14. Breast feeding status	17(56.7%)	5.7
15. Regularity of menstrual cycle	18(60%)	6.0
16. Abortion history	6(20%)	2.0
17. Current pregnancy status	21(70%)	7.0
18. Living children	12(40%)	4.0
19. Desire to have more child	5(16.7%)	1.7
20. History of contraceptive use	26(86.7%)	8.7
21. Current method use	20(66.7%)	6.7
22. History of pregnancy complications	8(26.7%)	2.7
23. Smoking status	0(0%)	0.0
24. History of STIs	0(0%)	0.0
25. Whether discussed contraceptives with partner(s)	11(36.7%)	3.7
26. Ease of returning to facility	30(100%)	10
27. Take blood pressure	2(6.7%)	0.7
28. Check weight	0(0%)	0.0
29. Check height	0(0%)	0.0
30. Explain the procedure to the client	16(53.3%)	5.3
31. Wash his or her hands before the exam	3(10%)	1.0
32. Wash his or her hands after the exam	3(10%)	1.0
33. Wear sterile gloves during the exam	13(43.3%)	4.3
34. Discuss return visit	30(100%)	10
35. Give appointment card	30(100%)	10
Total		167.1/350

### Mechanism to encourage continuity

To assess mechanism to encourage continuity, client-based mechanism of follow-up was assessed. Client-based mechanism of follow-up assessed were providing appointment card and information when to return. The assessment showed that all clients had follow-up appointment card and were told where to go for resupply. But exit interview showed that information on what to do if problem arise was given only to 19 (10.5%) clients.

### Constellation of service

According to Bruce, constellation of service is the way how service is organized and convenient to the clients. It is least universal and most conditioned by context [15]. In this case, we considered satisfaction, service integration, accessibility and average client waiting time.

### Satisfaction

More than half of clients, 107 (59.1%), were very satisfied and 70 (38.7%) were satisfied with the service they were using while only 2 (1.1%) were dissatisfied and the same number of clients 2(1.1%) were very dissatisfied with the service and mentioned waiting time as cause of their dissatisfaction.

Eight (80%) providers were satisfied with service they were providing but 2(20%) were not satisfied. The cause of dissatisfaction for one provider was management while other provider associated dissatisfaction with poor performance saying:

*“Since I’m serving community, my satisfaction is when community awareness is changed and improved; but little is done within community, so I’m dissatisfied”.* (Male, clinical nurse, 10 years’ experience)

### Service integration

About one-fourth of clients, 47(26%), used integrated services from which one client received TT vaccination and 46 clients were tested for HIV. But from 181 clients, 142 (78.5%) said that they received TT vaccination in past and 154 (85.1%) reported that they were tested for HIV/AIDS.

Integration of FP service with OPD, STI treatment, VCT, safe abortion, EPI, post abortion care and PNC were assessed by providers’ interview. Three services (PNC, EPI and post abortion care) were reported as integrated by more than half of providers.

### Geographical accessibility

About three fourths (77.4%) of clients reported that they had walked less than 1 h to reach HC while 14.4, 7.7 and 0.5% said that they had walked 1–2 h/s, 2–3 h and more than 3 h respectively. Mean walking time was 51.5 min (SD = 47.78).

### Waiting time

The average waiting time was 10.38 min (SD = 4.8). About three fifths, 115(63.5%), of clients waited less than ten minutes while the rest 66(36.5%) waited for 10–20-min to see provider.

### Overall judgment

As it was presented in above section, availability dimension was found 64% while compliance was 67%. The overall program implementation level was 66.6% and was found good as per agreed criteria (Additional file 1 and Table 1).

## Discussion

This process evaluation assessed program readiness and program process quality. We measured program performance as per stakeholders agreed criteria. The measurement shows that the program seems “good” as per agreed judgment matrix but notable gaps were presented as per quality of care.

Both health centers had working toilet, electricity, waiting room, and pipe water. But some of basic materials for service provision were lacking in both HCs. To mention few examination table, examination light, disinfectant solution and BP-apparatus were lacking in Asendabo HC while hand washing water, examination light and weight-height scale were lacking in Nada HC. These materials are very crucial for screening (assessing eligibility criteria) for contraceptive methods, following aseptic procedures and assessing for any medical conditions. The unavailability of these materials can affect clinical quality of care as presented under compliance.

Majority of clients (78.4%) used injectable method, followed by pills (12.2%). Even though there was slight change over time, method preference was similar with previous studies conducted in study zone [21], study region [22] and other region in the country [23]. Better progress was seen in terms of informed choice as previous study done in the study zone [21] showed that 21.1% of clients used methods that were not their choice while only 2.2% clients used methods that were not their choice in this case; this might be due to investments done in improving program and increasing community awareness.

Choice of contraceptive methods is a key element of high quality services that benefits both clients and programs. Clients benefit because they are able to select the method that best meets their needs and can switch to a different method as their needs change or if they experience difficulties which is influenced by personal concerns, health considerations, cost, and the cultural environment. Programs benefit as clients are more likely to be satisfied and continue using a method [24]. Unless providers discuss methods to users, clients might not choose methods that are not common or that they do not know. During observation, majority of clients (93.3%) were informed about injectable while only about half (53.3%) heard about implants. With client exit interview, only less than half of clients (44.2%) reported that possibility of changing method was discussed. In case when problem arises, these clients might stop using contraceptive without trying another contraceptives.

Basic information that should be provided for family planning service users in relation to chosen methods was assessed using client-exit interview and observation and there were some differences between results of two data collection methods (Fig. 3). These results again differed from HC to HC except HIV/AIDS prevention which was zero for both HCs with two data collection techniques. Data collected from exit interview showed higher figure of information on how to use and advantages than observation and these might be due to clients’ past knowledge report because majority of service users were continuing users and they know at least how to use and advantages. But disadvantages, possible side effects, what to do if problem arise and possibility of changing method were higher with observation which might be due to providers’ behavioral change of performance during observation as different studies reported that side effects and disadvantages are not discussed during routine service provisions [21, 22].

Under technical competency, assessment of clinical techniques that should be performed showed poor performance needing improvement measures. Some of critical performances included measuring height and weight (0%), asking history of STI (0%), asking history of smoking (0%), measuring blood pressure (6.7%) and counselling desire to have more children (16.7%). This was much lower than reports of studies done in similar zone [21] and in other region [25]. During assessment of availability of materials for quality service provision, it was noted that there was shortage of BP apparatus and though there were trained providers, most sessions were provided by untrained providers. While majority of clients missed important services, they were satisfied with the service which could be influenced by their awareness of clinical standard and rural context as satisfaction is more contextual than universal.

The mechanism to encourage continuity of service includes mass media and client-based tools, but here only client-based mechanism especially client follow-up (client card and information provided when to return) was assessed. The assessment with both observation and exit interview showed that in both HCs, all clients had follow-up card and with observation, all clients were told where to go for resupply. Counseling what to do if problem arise is good way to encourage clients but only 19 (10.5%) clients heard what to do if problem arise.

Government of Ethiopia is doing much to make FP accessible through health extension program by which health extension workers are assigned in health posts at Kebele level (smallest administrative structure). As result, majority of clients (77.3%) traveled less an hour to get service. In addition, the average waiting time was also 10.38 min though it was cause for dissatisfaction of some clients (2.2%).

## Conclusion

Although clients were satisfied and program implementation level was good according to stakeholders agreed judgments, several deficiencies like unavailability of crucial materials, poor technical performance including poor information provision, poor integration of services, and failure to follow infection prevention procedures were noted. Crucial materials for provision of quality service should be availed and providers should strictly adhere to clinical guidelines. Quality of process should be monitored to improve service by integrating existing services in resource limited setting.

## Additional file

Additional file 1: Evaluation dimension, indicators and source of data for evaluation of FP program, Omo Nada district, 2011. (DOCX 17 kb)

## Abbreviations

AIDS: Acquired Immune Deficiency Syndrome
EPI: Expanded Program on Immunization
FP: Family planning
HC: Health Center
HIV: human immunodeficiency virus
IEC: Information, Education and Communication
IUCD: Intrauterine Contraceptive Device
OPD: Out Patient Department
PNC: Postnatal Care
RH: Reproductive Health
SPSS: Statistical Package for the Social Sciences
STI: Sexually Transmitted Infections
TT: Tetanus Toxoid
VCT: Voluntary Counselling and Testing
