# Supplementary material for: Missed opportunities in family planning: process evaluation of family planning program in Omo Nada district, Oromia region, Ethiopia
Source: Contracept Reprod Med. 2018 Aug 1;3:14. doi: 10.1186/s40834-018-0068-7 (PMC6069553; doi:10.1186/s40834-018-0068-7)
Supplement: Supplementary file 1 — Evaluation dimension, indicators and source of data for evaluation of FP program, Omo Nada district, 2011. (DOCX 17 kb) [file 40834_2018_68_MOESM1_ESM.docx]

Evaluation dimension, indicators and source of data for evaluation of FP program, Omo Nada district, 2011

| **Sub-dimensions** | **Items to be evaluated** | **Indicators/items measured** | **Weight** | **Source of data** |
| --- | --- | --- | --- | --- |
| Availability | Availability of trained provider, contraceptives, amenities, IEC materials | 1. Contraceptives (6 items), trained provider, amenities (13 items), IEC materials (5 items)   (Each item out of 10) | 250 | Observation  Provider interview  Observation |
| Information to clients | Information provided to clients | 1. Proportion of clients who received possible side effects about method | 100 | Exit interview |
| Technical competence | Providers’ clinical techniques | 1. Appropriate use of clinical guidelines (35 items from greeting to giving appointment) (Each item out of 10) | 350 | Observation |
| Interpersonal relation | Provider-client interaction and privacy | 1. Proportion of FP users who report freely expressed their opinion 2. Proportion of FP users who feel all procedures are private | 200 | Exit interview |
| Mechanism for encouraging | Continuity of program use | 1. Proportion of FP users who have appointment card 2. Proportion of clients who were told where to go for resupply 3. Proportion of clients who have got information on what to do if problem arise | 300 | Exit interview |
| Appropriate constellation of service | Satisfaction | 1. Proportion of FP users who are satisfied with service 2. Proportion of FP providers satisfied with service they giving | 200 | Exit interview  Provider interview |
|  | Geographical accessibility | 1. Proportion of FP users who have walked less than or equal to 2hrs to HC | 100* | Exit interview |
|  | Integration of service | 1. Integration of FP with other department | 70** | Provider interview |
|  |  | 1. Proportion of FP users who got TT vaccination 2. Proportion of FP users counseled and tested for HIV | 200 | Exit interview |
|  | Waiting time | 1. Average waiting time of FP clients to see providers | 30*** | Exit interview |

* - access to HC was considered because at the time of study, implants insertion and removal was done at HC

** - service was counted when reported by more than half of participating providers

*** - average waiting time less than 15min = 30; 15-30 min. = 20; 30min-1hour = 10 and 1 hour = 0

Judgment matrix used to evaluate FP program, Omo Nada district, 2011

| **Items to be evaluated** | **Indicators/items measured** | **Weight** | **Observed** | **Remarks** |
| --- | --- | --- | --- | --- |
| Availability of trained provider, contraceptive, amenities, IEC materials | 1. Contraceptives (6 items), trained provider, amenities (13 items), IEC materials (5 items)   (Each item out of 10) | 250 | 160 (64%) | 4 contraceptive options, trained provider, 9 amenities and 2 IEC materials seen |
| Information provided to clients | 1. Proportion of clients who received possible side effects about method | 100 | 20.4 |  |
| Providers’ clinical techniques | 1. Appropriate use of clinical guidelines (35 items from greeting to giving appointment) (Each item out of 10) | 350 | 167.1 | Proportions were calculated out of 10 and summed |
| Provider-client interaction and privacy | 1. Proportion of FP users who report freely expressed their opinion 2. Proportion of FP users who feel all procedures are private | 200 | 147.5 | 64.1% clients expressed idea freely; 83.4% clients reported privacy was maintained |
| Continuity of program use | 1. Proportion of FP users who have appointment card 2. Proportion of clients who were told where to go for resupply 3. Proportion of clients who have got information on what to do if problem arise | 300 | 210.5 | 100% clients had client card, 100% were told where to go for resupply and 10.5% clients heard what to do if problem arise |
| Satisfaction | 1. Proportion of FP users who are satisfied with service 2. Proportion of FP providers satisfied with service they giving | 200 | 177.8 | 97.8% clients were satisfied; 80% providers were satisfied |
| Geographical accessibility | 1. Proportion of FP users who have walked less than or equal to 2hrs to HC | 100 | 91.8 |  |
| Integration of service | 1. Integration of FP with other department | 70 | 30 | 3 services were integrated |
|  | 1. Proportion of FP users who got TT vaccination 2. Proportion of FP users counseled and tested for HIV | 200 | 163.6 | 78.5% of clients got TT vaccination; 85.1% clients were tested for HIV |
| Waiting time | 1. Average waiting time of FP clients to see providers | 30 | 30 | Average waiting time was <15 min (10.38min) |
| Compliance total | | 1550 | 1038.7 (67%) |  |
| **Total** | | **1800** | **1198.7** | **66.6%** |
